# Metagenomics of rumen bacteriophage from thirteen lactating dairy cattle

**DOI:** 10.1186/1471-2180-13-242

**Published:** 2013-11-01

**Authors:** Elizabeth M Ross, Steve Petrovski, Peter J Moate, Ben J Hayes

**Affiliations:** 1 Department of Environment and Primary Industries, Bundoora, VIC 3086, Australia; 2 Dairy Futures Cooperative Research Centre, Bundoora, VIC 3086, Australia; 3 La Trobe University, Bundoora, VIC 3086, Australia

**Keywords:** Virome, Rumen, Bacteriophage, Metagenomics

## Abstract

**Background:**

The bovine rumen hosts a diverse and complex community of *Eukarya, Bacteria, Archea* and viruses (including bacteriophage). The rumen viral population (the rumen virome) has received little attention compared to the rumen microbial population (the rumen microbiome). We used massively parallel sequencing of virus like particles to investigate the diversity of the rumen virome in thirteen lactating Australian Holstein dairy cattle all housed in the same location, 12 of which were sampled on the same day.

**Results:**

Fourteen putative viral sequence fragments over 30 Kbp in length were assembled and annotated. Many of the putative genes in the assembled contigs showed no homology to previously annotated genes, highlighting the large amount of work still required to fully annotate the functions encoded in viral genomes. The abundance of the contig sequences varied widely between animals, even though the cattle were of the same age, stage of lactation and fed the same diets. Additionally the twelve animals which were co-habited shared a number of their dominant viral contigs. We compared the functional characteristics of our bovine viromes with that of other viromes, as well as rumen microbiomes. At the functional level, we found strong similarities between all of the viral samples, which were highly distinct from the rumen microbiome samples.

**Conclusions:**

Our findings suggest a large amount of between animal variation in the bovine rumen virome and that co-habiting animals may have more similar viromes than non co-habited animals. We report the deepest sequencing to date of the rumen virome. This work highlights the enormous amount of novelty and variation present in the rumen virome.

## Background

The value of domestic ruminants (cattle, sheep and goats) comes from their ability to convert, by means of rumen fermentation, low-quality forages into high quality, high protein products (milk and meat) suitable for human consumption [1]. The rumen, the first section of the ruminant stomach, contains symbiotic microorganisms that breakdown ingested food. These microorganisms include *Eukarya, Bacteria, Archaea*[2], and large numbers (>10^7^ pfu ml^-1^) of virus including bacteriophage [3]. The entire bacteriophage or 'phage’ population in a sample is termed a virome.

Massively parallel sequencing (MPS) is a technology which allows the generation of several million DNA sequences in parallel. MPS has been used to investigate the *Bacterial* and *Archaeal* populations of the bovine (*Bos taurus*) rumen [4-7]. However, MPS studies on the rumen virome are scant. One study [8] sequenced 425 Mbp of viral DNA from the rumen of three animals, one lactating, one non lactating and one culled animal. The MPS study [8] and earlier studies such as [9], both suggest that there are large amounts of variation in the bovine rumen virome. However some of the variation may have been due to external sources (diet, lactation state, location, time of sampling).

Since the recent rumen virome study [8], there have been technological advances that have allowed the volume of sequence data to increase by an order of magnitude. These advances now enable the sequencing of viromes from a greater numbers of animals, resulting in the ability to gain an insight into natural variation between animals. MPS virome studies have made it possible to study viral genomes even when the host bacterium cannot be cultured. However, there are significant challenges involving analysis of MPS virome data [10]. Tools for the analysis of virome samples [11-13] are becoming more readily available allowing estimates of virome characteristics such as species richness. The combination of published analysis techniques, along with the increased ability to generate data has allowed the field to switch from descriptive analyses to quantitative investigations of how various factors (such as host effects or temporal variation) affect the virome [14].

To access the amount of variation in the rumen virome we extracted virus like DNA from the rumen fluid of 13 lactating dairy cattle. Twelve of the 13 cows were housed together, fed the same diet, had a shared location and were sampled on the same day, allowing us to investigate the magnitude of natural variation in the rumen virome.

## Results and discussion

### Assembly and annotation

**Figure 1:**
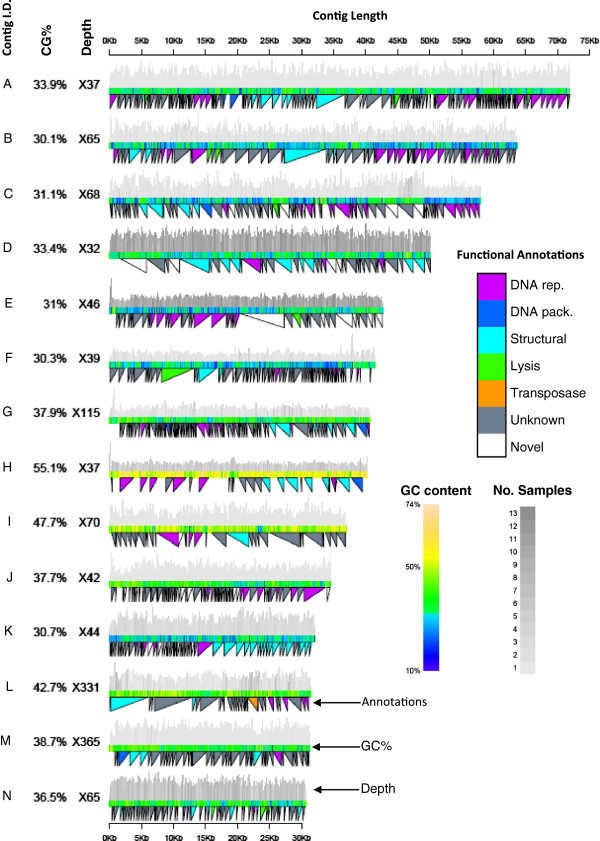
**Virome contigs greater than 30 kbp.** The median coverage of the contig and the GC% are shown to the left of each contig. The colour of the centre bar represents the GC% in a 100 bp sliding window. The grey lines on top of each contig represent the sequence depth, as determined by aligning reads to the contig using BWA. The shade of grey represents the number of different samples that have reads mapping to that position. The triangles below the contig show the function of the annotation at that position. DNA rep. = DNA replication and repair, DNA pack. = DNA packaging, Unknown = all significant BLAST hits were to unannotated subjects, Novel = no significant BLAST hit.

**Figure 2:**
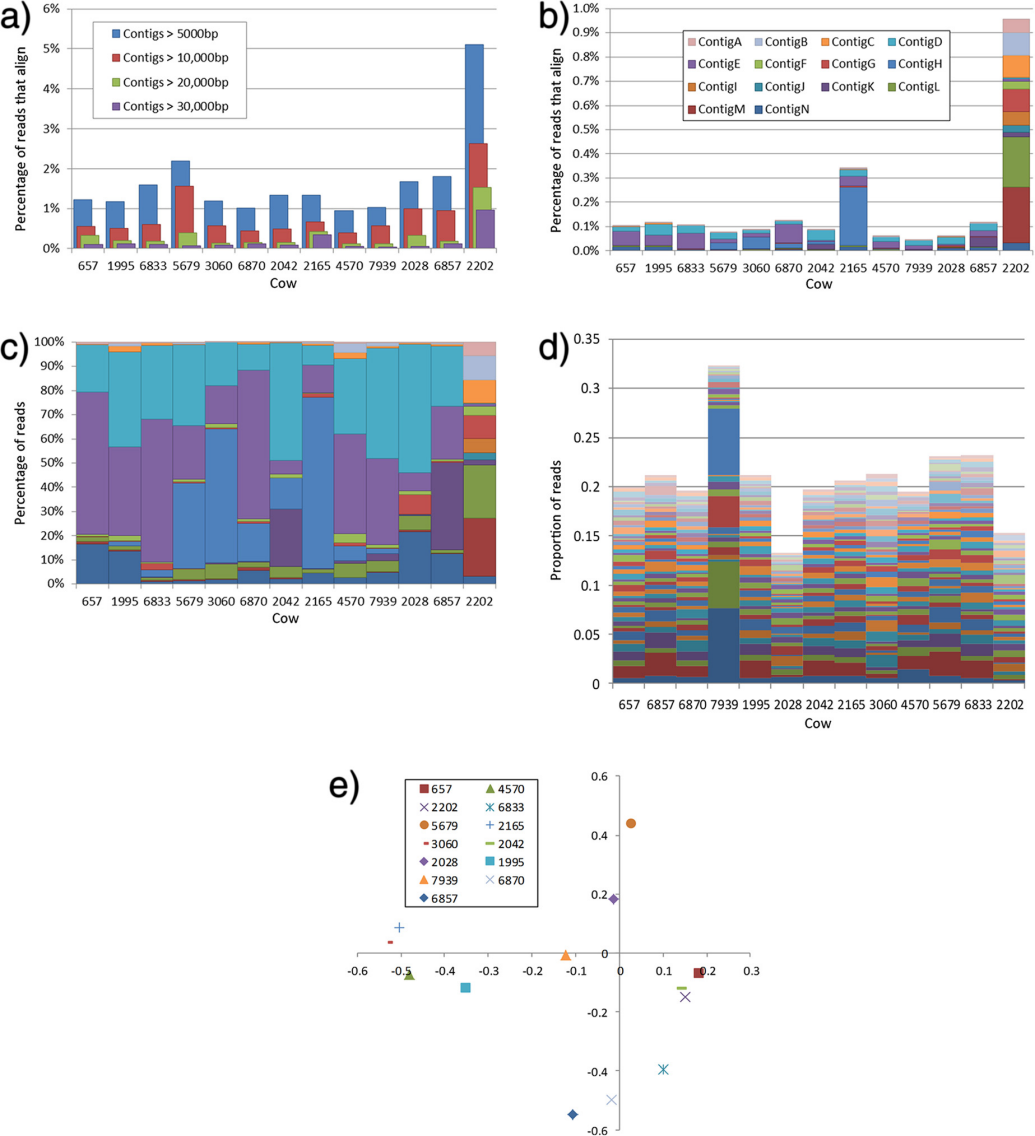
**Between animal variation in alignments to contigs. a)** Percentage of reads that map back to assembled contigs of different sizes. **b)** Distribution of reads that map to the 14 contigs annotated in Figure 1. **c)** Distribution of reads that map to the 14 contigs annotated in Figure 1 normalised to a total of 100%. **d)** Distribution of reads that map to the 100 most abundant (in our dataset) contigs assembled from [8]. **e)** Principle component analysis of reads that map to the 50 most abundant contigs assembled from [8]. PC1 (X axis) explains 15.42% of the variation, PC2 (Y axis) explains 12.73% of the variation.

We sequenced virus like particles from the rumen fluid of 13 lactating Australian Holstein dairy cattle on the HiSeq2000. Each library contained between 2 and 57 million read pairs (Table 1). After a combined assembly of all samples, 14 contigs were greater than 30 Kbp in length. These contigs were manually inspected for even coverage and annotated (Figure 1). Three of the contigs have all the genes necessary for a complete functional phage genome (contigs A, H and K). We mapped our sequence reads back to the complete set of contigs. While up to 57.5% of reads mapped to the whole assembly, only 0.84% of reads mapped to contigs greater than 10 Kbp (Figure 2a). This would suggest that although we have assembled up to 57% of the bovine rumen virome, most of what has been assembled is still highly fragmented, and contained in small contigs.

**Table 1:** Virome sequence volume, alignments and richness

		**Alignments to other viromes (%)**						**Richness by CatchAll**			
**Sample**	**Data trimmed**^**T **^**(Read pairs)**	**Cow 6993 **[8]	**Cow 7664 **[8]	**Cow 7887 **[8]	**Swine faeces **[18]^**+**^	**Human saliva **[19]^**+**^	**Pond **[20]^**+**^	**Best model**^**B**^	**Estimated total species ± S.E.**	**Best discounted model**^**D**^	**Estimated total species ± S.E.**
Cow 2202^C^	52,239,452	3.22	3.25	1.86	0.07	<0.01	<0.01	2MixedExp	5550786 ± 465864.1	SingleExp	18412.6 ± 1544.2
Cow 657	2,116,650	4.86	4.91	3.12	0.05	<0.01	<0.01	3MixedExp	4679342 ± 676180.6	2MixedExp	26795.8 ± 3871.9
Cow 1995	3,410,438	3.88	4.69	2.69	0.04	<0.01	<0.01	2MixedExp	5952188.7 ± 295239.5	SingleExp	5313.4 ± 263.5
Cow 2028	6,956,332	3.57	4.24	2.30	0.04	<0.01	<0.01	3MixedExp	6515260.9 ± 338382	2MixedExp	3369.8 ± 175
Cow 2042	4,048,214	3.19	3.71	2.22	0.03	<0.01	<0.01	2MixedExp	7904757.2 ± 622624.3	SingleExp	9023.1 ± 707.6
Cow 2165	4,362,890	4.57	5.14	3.20	0.04	<0.01	<0.01	4MixedExp	10353681.8 ± 119769.7	3MixedExp	1376492.9 ± 15923
Cow 3060	3,899,392	4.53	5.01	3.35	0.05	<0.01	<0.01	2MixedExp	5805999.9 ± 388938	SingleExp	11428.7 ± 765.5
Cow 4570	4,365,420	3.41	4.05	2.53	0.04	<0.01	<0.01	4MixedExp	22410379.6 ± 511083.2	3MixedExp	4126756.3 ± 94113.3
Cow 5679	3,681,922	3.70	4.84	2.67	0.04	<0.01	<0.01	3MixedExp	6098575.2 ± 404293.2	2MixedExp	6772.1 ± 448.5
Cow 6833	3,455,882	3.58	4.18	2.55	0.05	<0.01	<0.01	3MixedExp	7427673 ± 2447886.4	2MixedExp	30174.7 ± 9943.8
Cow 6857	8,998,900	4.18	4.71	2.77	0.04	<0.01	<0.01	3MixedExp	7916340 ± 2025360.6	2MixedExp	23466.2 ± 6003
Cow 6870	3,906,564	4.49	5.88	3.60	0.03	<0.01	<0.01	2MixedExp	4794540.3 ± 304303.4	SingleExp	13438.6 ± 852.7
Cow 7939	5,119,322	3.45	5.02	2.84	0.03	<0.01	<0.01	2MixedExp	6757358.4 ± 455261	SingleExp	7504.3 ± 495.6
Average*	8,197,029	3.89	4.59	2.75	0.04	<0.01	<0.01	-	7,858,991	-	435,304

*Average of all samples from this study.

^C^Cannulated animal.

^T^Amount of sequence remaining after removal of poor quality bases.

^+^Multiple samples from study combined.

^B^Best Model as assessed by the CatchALL program.

^D^Best Model that has been corrected using the assumption of overrepresented singletons.

Five of the 14 largest contigs were observed in all samples (≥10 reads mapping back to contig), with all 14 observed in the sample from animal 2202 (which had the deepest sequence coverage; (Table 1). Despite over a third of the most common contigs being represented in all samples, the most abundant contig only accounted for 0.24% of sequence reads (contig H in animal 2165; Figure 2b). There was a strong correlation between the sequencing depth of the sample and the percentage of reads that mapped back to the assembly (contigs > 5 Kbp; r = 0.952), partly reflecting the fact that the contigs were assembled from the same data. As sequence depth has a strong impact on contig size, it is likely that we have only assembled genomes from the most dominant phage species in the bovine rumen.

Of the 815 putative genes in the 14 largest contigs, 61.5% were assigned an unknown function because all of the significant BLAST hits were themselves unannotated. Genes involved with either DNA replication or repair consisted 13.3% of putative genes, 9.2% of putative genes were structural and 1.8% appear to be involved in lysis. Completely novel (no significant BLAST hits to the public nt database) putative genes made up an additional 13.0%. These novel and unannotated genes may encode unknown functions including those involved in cell lysis or lytogenic reproduction of the phage.

Phage may be integrated into the genomes of their host prokraryotes. To look for evidence of this we aligned sequence data from the microbiomes of the same samples from [15] to the 14 largest contigs. All of the largest contigs were observed in one or more microbiome samples. This could be explained by viral contamination of the microbiome. Such contamination would result in the same proportions of viral contigs in the microbiome as are observed in the virome. Therefore the proportions of reads that map to the viral contigs in the microbiome were compared to the virome. The abundance of two contigs, E and M, were significantly higher in the microbiome than expected under the viral contamination model (p < 0.05 after a Bonferroni correction). This is evidence that these two phage are integrating into the prokaryote genomes. However this hypothesis that cannot be validated until the host genomes are sequenced or the host and phage and isolated and cultured. Additionally, contigs A, B, C, E, F, G and N show homology to genes involved in lysis, suggesting that they may be lytic, however this is again speculation until the phage are isolated.

### Variation in contig abundance between animals

The twelve co-habited animals’ viromes appear to be dominated by contigs D, E and J, while animal 2202’s virome shows a much more even coverage between contigs (Figure 2b). However these contigs only account of <0.34% of the viromes of the co-habited animals. There also appears to be a heterogeneity in the distributions of contigs between animals (Figure 2b-c). Contig H has a high abundance in cow 2165 (10,480 reads mapped back to the contig), but is almost below detectable limits in the deepest sequenced animal (2202, only 10 reads mapped back to the contig) and is undetectable in cow 657 (0 reads mapped back to the contig). The evenness observed in the virome of animal 2202 possibly hints that the rumen viral community is not dominated by a single species, but has a large number of taxa with a reasonably even distribution. This evenness of distribution was likely not observed in our other samples due to their limited sequence depth resulting in many unassembled species. To assess the distribution on an independent set of contigs we aligned our reads to contigs assembled from the independent set of animals from [8]. Between 0.22% and 0.70% of our reads aligned to the contigs. The distribution of contigs was much more even when these independent contigs were used (Figure 2d). A principle component analysis did not separate the independently housed animal from the co-house animal, although as less than 1% of the reads from any virome aligned, this should be interpreted with caution. The evenness of the phage population concurs with the findings of [8] that even the most abundant phage species represent only a small proportion of the rumen virome. Our finding that the most common of phage contigs only represent 0.24% of the virome is consistent with this. The higher degree of similarity between the viromes of the 12 co-habited animals and animal 2202 may suggest that there is transfer of viral species between the hosts within a herd, although 2202 was also cannulated, this confounding effect is not possible to resolve without further virome data. Still the variation between co-habited animals (for example contig H in animals 2165 and 657) illustrates that any transfer between animals does not maintain a completely homogeneous herd. Whether the within herd variation is related to traits that are related to rumen microbiome functions, such as methane emission levels or bloat susceptibility is yet to be seen, however this dataset shows that there is natural within herd virome variation which should be investigated. Future studies may explore the relationship between this variation and host traits such as disease susceptibility and methane emissions.

### Species richness

Based on our sequence data, it is clear that estimations of rumen phage diversity using electron microscopy of less than 40 species per host summerised in [16] are dramatic underestimations, and the true number of species likely lies closer to the estimate in [8] of between 4 and 27 thousand. To estimate the number of species present in each of our samples, we used CatchAll [13]. The estimated total number of species were between 4.6 and 22.4 million per sample (Table 1). Over estimations of richness can be caused by spurious singletons, and the biological and technical causes for these have been outlined in [13]. To account for spurious singletons we also used a model which corrects for such artefacts. This model reported between 3,370 and 4,126,756 (Table 1) species per sample. The divergence between these results and those reported in [8] is not surprising given that the authors of CatchAll consistently report richness higher than PHACCS [12], and claim that PHACCS underestimates richness. Our assembly contains 272 Mbp of assembled sequence, if we assume an average phage genome size of 30 Kbp this results in approximately 9073 genomes (assuming little overlap between contigs) worth of sequence in our assembly. Given that we have only assembled half of the sequence in our samples, and the assembled half would be the less diverse than the unassembled half, approximately 18 thousand genomes could be expected as a lower bound for number of species estimations. This rough calculation concurs with the CatchAll discounted model estimate for cow 2202, and together they tend to suggest that previous assessments of rumen phage richness are indeed underestimations. In any case, it is clear that there is a large amount of variation in the species that make up the *B. taurus* rumen virome. Also the large range of estimates, even under the reduced model, suggests that the viromes of some animals are much more species rich than others. This could be an indication of a more diverse prokaryote population in some animals than others. This large range should be considered in future experimental designs, as deeper sequencing of particularly diverse samples will be needed to capture a snapshot of the viral community.

### Comparison to other viromes

There are a number of host associated and non host associated viromes sequenced to date. To compare these virome sequence profiles to ours we obtained reads from publically available datasets derived from published studies. Here we have only isolated viral DNA (not RNA), therefore we were unable to compare our results to studies such as that by [17], which isolated RNA viruses. We selected four different types of viromes for comparison with our data. The viromes included samples from cattle rumen, human saliva, swine faeces and non host-associated environments [8,18-20]. The reads from the published viromes were aligned to our contigs. The other bovine rumen viromes had only a small percentage (between 0.70% and 1.56%; Table 2) of reads that mapped to our contigs. When we compared our reads to reads in the virome obtained from the rumen fluid of cattle as previously reported [8] only 2-5% of our reads mapped (Table 1). This would suggest that there are substantial differences between the viromes observed by [8] and ourselves. These differences could be a result of the phage isolation technique [21], or possibly the sequencing technology used. It is also possible that the differences were due to real variations, as there was large amounts of variation observed within our samples and it could be expected that cows from other locations and on different diets might be even more distinct.

**Table 2:** Virome alignments to assembled contigs

**Sample**	**All contigs**	**Contigs >5000 bp**	**Contigs >10,000 bp**	**Contigs >20,000 bp**	**Contigs >30,000 bp**
Average Rumen Virome*	37.1%	1.66%	0.84%	0.33%	0.17%
Cow 6993 [8]	0.70%	0.03%	0.01%	<0.01%	0.00%
Cow 766 [8]	0.56%	0.03%	0.01%	<0.01%	<0.01%
Cow 7887 [8]	1.56%	0.03%	<0.01%	0.00%	0.00%
Swine Faeces [18]^+^	0.01%	<0.01%	<0.01%	0.00%	0.00%
Human Saliva [19]^+^	0.00%	0.00%	0.00%	0.00%	0.00%
Pond [20]^+^	<0.01%	0.00%	0.00%	0.00%	0.00%

^+^Multiple samples from study combined.

*Average of all samples from this study.

We then compared our reads to the non-bovine viromes. The swine faeces virome [18], the human saliva virome [19] and aquaculture pond derived virome [20] did not have any reads mapping to our contigs (Table 2). We then attempted to map our reads to the 454 reads of the above mentioned databases. Less than 0.1% of our reads mapped to the swine faeces database, while less than 0.01% of our reads mapped to the human saliva or pond viromes (Table 1). There is an observable cline in the amount of our reads that map to these databases as they become less similar to our sample type, from highest from our own assembly, followed by the same sample type (bovine rumen), then an alternative species digestive tract (swine faeces), then non gut derived viromes.

This cline in reads that align to contigs from different habitats may be a result of the similar prokaryote populations within gut samples. Both swine faeces and bovine rumen are dominated by Firmicutes and Bacteroidetes phyla [6,22], while the salivary microbiome is dominated by Proteobacteria [23]. Hence the viral species in gut samples likely prey mostly on Firmicutes and Bacteroidetes, which would result in more similarities between the gut derived viromes then between the rumen and non-gut derived viromes.

### Functional characteristics of the rumen virome

**Figure 3:**
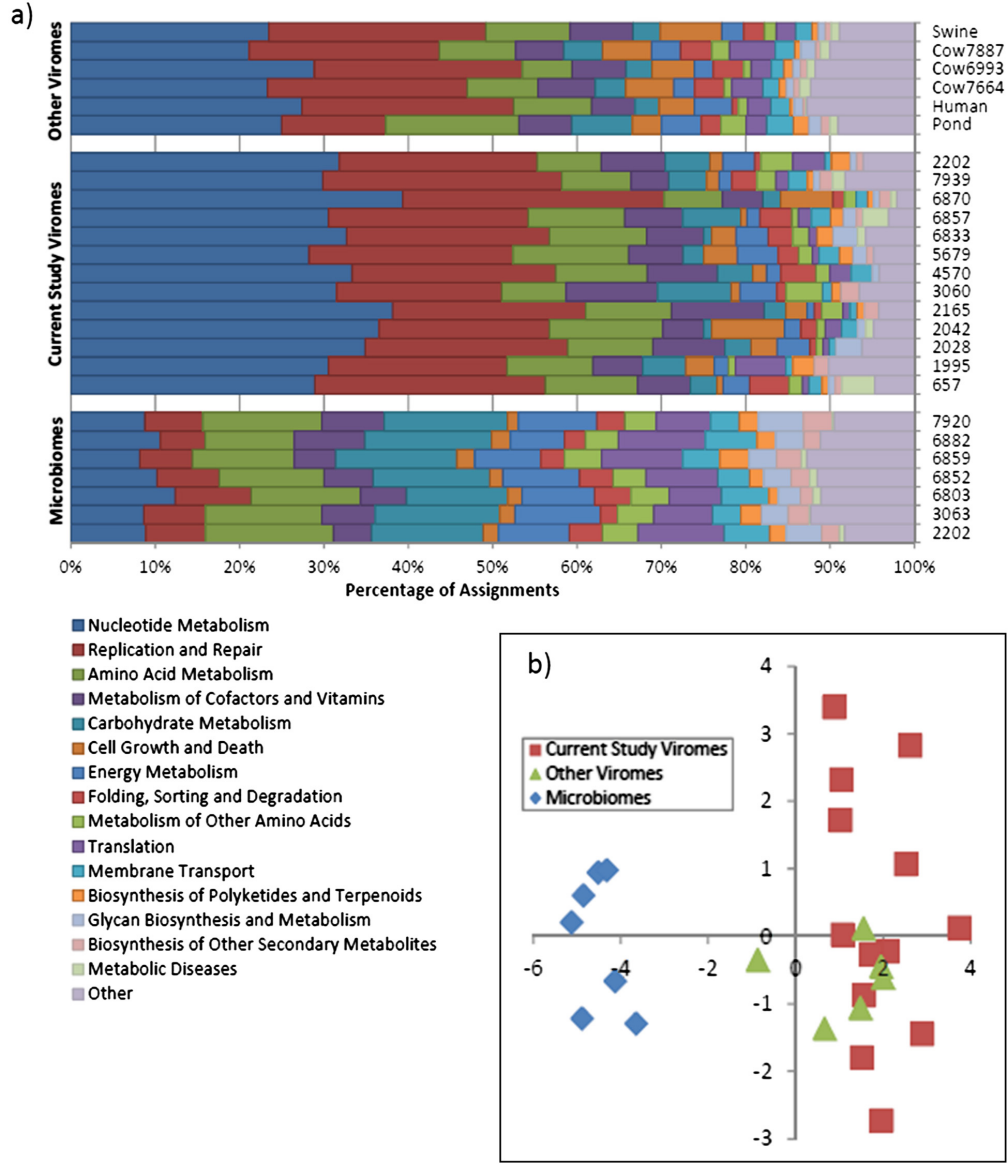
**Between animal variation in KEGG pathways. a)** Second level KEGG assignments of sequence reads. The “microbes” samples are microbial metagenomes [6]; other viromes include representatives from cattle, human saliva, swine faeces and non host-associated environments [8,18-20]. **b)** Principle component analysis of the second level KEGG pathways. PC1 (X axis) explains 53.1% of the variation, PC2 (Y axis) explains 13.1% of the variation.

We also investigated the hypothesis that the functional characteristics of the bovine rumen virome were conserved between animals despite the large amount of sequence variation observed. We assigned out reads to KEGG second level pathways. Of the assigned second level KEGG pathways, Nucleotide Metabolism and Replication and Repair combined made up 57% of virome assignments. This is in contrast to the prokaryote metagenomes of rumen fluid from animals from the same location [6] (Figure 3a). Evenness of second level KEGG pathways was significantly lower in the phage sequences compared to the microbe data (t-test, p < 0.001; Table 3). This unevenness in the phage dataset is likely due to the lack of genes coding for functions such as carbohydrate metabolism, which the phage relies on the host cell to provide. We then compared the functional characteristics of our sequences to other viromes. A principle component analysis revealed that all viromes clustered, and all microbial metagenomes clustered based on assignments to KEGG second level pathways (Figure 3b). These analyses reveal how the virome of a sample is functionally distinct from the microbial metagenome, and that the functional characteristics of viromes are more similar to each other than to microbial metagenomes, even when the viromes are taken from different sample types (e.g. aquaculture ponds versus rumen fluid). This is likely a reflection of the limited number of annotatable functions encoded for in the phage metagenome. Interestingly the functions coded for in our viromes are not as variable as that observed in human faeces [14]. Some of the human faeces variation may be caused by the small number of reads which could be used in the KEGG analysis due to the limited percentage of alignments and the starting data volume.

**Table 3:** Diversity and evenness of KEGG pathways

**Metagenome source**	**Sample**	**Metagenome type**	**Categories observed**^**N**^	**Shannon diversity Index (H’)**	**Evenness (E)**
This study	Cow 2202^C^	Virome	20	2.192	0.732
	Cow 657	Virome	21	2.162	0.710
	Cow 1995	Virome	22	2.299	0.744
	Cow 2028	Virome	20	2.052	0.685
	Cow 2042	Virome	17	2.011	0.710
	Cow 2165	Virome	16	1.911	0.689
	Cow 3060	Virome	16	2.150	0.775
	Cow 4570	Virome	15	2.023	0.747
	Cow 5679	Virome	18	2.184	0.756
	Cow 6833	Virome	19	2.125	0.722
	Cow 6857	Virome	18	2.142	0.741
	Cow 6870	Virome	16	1.756	0.633
	Cow 7939	Virome	21	2.200	0.722
	Average*	Virome	18.4	2.093	0.720
Other Studies	Cow 6993 [8]	Virome	36	2.425	0.677
	Cow 766 [8]	Virome	34	2.553	0.724
	Cow 7887 [8]	Virome	35	2.580	0.726
	Swine Faeces [18]^+^	Virome	33	2.398	0.686
	Human Saliva [19]^+^	Virome	36	2.221	0.620
	Pond [20]^+^	Virome	31	2.537	0.739
	Cow 2202^C^[6]	Microbial	27	2.731	0.829
	Cow 3063^C^[6]	Microbial	30	2.794	0.822
	Cow 6803^C^[6]	Microbial	30	2.778	0.817
	Cow 6852^C^[6]	Microbial	29	2.809	0.834
	Cow 6859^C^[6]	Microbial	29	2.860	0.849
	Cow 6882^C^[6]	Microbial	24	2.752	0.866
	Cow 7920^C^[6]	Microbial	28	2.762	0.829

*Average of all samples from this study.

^C^Cannulated animal.

^N^Number of second level KEGG categories observed in sample’s data.

H’ – Shannon diversity Index.

E – Evenness (H’ / H_max_).

^+^Multiple samples from study combined.

The relatively narrow functional characteristics observed in the rumen fluid virome may be in part due to the lack of homology to annotated genes in the databases used for annotation (Figure 1). The potential of novel bacteriophage derived genes to control the bacterial populations of the host will likely be of interest in both a biomedical sense (for diseases which are associated with bacterial populations such as inflammatory bowel disease) and, in the case of livestock, reducing enteric methane emissions from the rumen.

### Rumen virome importance

Domestic ruminants are important production animals to people worldwide. Their ability to use microbial fermentation to provide energy for food (meat and milk), fibre (wool) and power (bullocks used as draught animals) has seen their numbers soar to 3.6 billion [24]. Because of this, researchers have long been interested in the rumen microbiome, however the virome has been neglected due to the lack of appropriate marker genes and analysis benchmarks. It is imperative that the virome is studied along with the microbiome and the host animal. The virome, being pathogens of the microbiome, can substantially alter rumen function, which in turn may hinder the ability of the host to utilise feed efficiently. Additionally, as the microbiome in ruminants is much more closely linked to major nutrient acquisition than in humans and mice, a manipulation of the virome (and therefore the microbiome) may enable a more substantial manipulation of nutrient acquisition than is possible in monogastric animals.

Phages have been suggested as a possible methane mitigation strategy through “bacteriophage therapy” [25]. This strategy requires identification of phage species which target the dominant methane producing Archaea in the rumen. Such approaches will benefit from metagenomic virome sequencing by the availability of additional information regarding the dominant phage species in the rumen virome, and will help establish an understanding of the stability or variability of the rumen virome over time, which may affect such therapies. Furthermore, comparison of the virome and microbiome of each rumen sample may shed light on the ecological relationship between phage and bacterium. For now, this work has illustrated the vast amount of species richness and between animal variation in the rumen virome. Identifying which of these sequences may belong to Archaea pathogens is a significant challenge for future work.

Studies in other species have found that phages can have a protective role for their host [26], and studies investigating control of bacterial gut populations with phages have been successful [27]. The rumen virome of cattle may be the key to unlocking the secretes of the optimally functioning rumen, and therefore, should be a consideration in the quest to develop the ultimate production animal.

## Conclusions

We have completed the deepest sequencing of the rumen virome to date. The results indicate large taxonomic diversity between the rumen viromes of Australian Holstein dairy cattle, and that animals housed together may have more similar viromes than those housed separately. However, this taxonomic variation is not reflected in the functional characteristics of the rumen virome. In fact, the rumen virome appears to be functionally conserved between animals. This suggests that while rumen viral genome sequences diverge rapidly between hosts, functional characteristics are under significant evolutionary constraint.

The observed differences between the rumen virome and the rumen microbiome likely reflect the much narrower set of functions typically encoded in a viral genome compared to a microbial genome. As MPS becomes more widely used to investigate viromes, it will be interesting to observe how these species correlate with changes in the bacterial population, or even if population differences are associated or predictive of key traits such as feed conversion efficiency and methane yield.

## Methods

### Animals

Animals used in this study have been previously described [6,28]. All animals were adult lactating Holstein dairy cattle. Twelve of the 13 cows were house together, fed the same diet, had a shared location and were sampled on the same day, the remaining cow (2202) was a cannulated individual which had been five months earlier. Rumen fluid from animal 2202 was removed via the fistula, rumen fluid from all other animal was collected via stomach pump. All work involving animals was approved by the Department of Environment and Primary Industries Animal Ethics Committee.

### Molecular techniques & sequence quality control

A cell pellet was removed from the rumen fluid via centrifugation and then viral DNA was extracted from the supernatant as per [29]. The putative phage DNA was sequenced on the Illumina HiSeq2000 on a 101 bp paired end run.

Poor quality sequence was removed from the dataset using dynamic trimming such that no read had an average Phred quality score below 20, and no read contained more than three bases with a Phred quality score below 15.

### Assembly and annotation

Sequence from all animals was computationally pooled and assembled into contigs using Gossamer [30]. Because of the complexity of metagenome samples, several assemblies were performed at different K-mer lengths. These assemblies were then further combined in a single Gossamer assembly (K-mer = 51). The 14 contigs that were greater than 30 Kbp were inspected for even coverage and manually annotated. Annotation was performed using Integrative services for genomic analysis [31] and the BLASTp algorithm at NCBI to determine the presence of conserved motifs. The majority of genes could not be assigned a function, either because all hits with significant homology were either unannotated or predicted proteins, or because the open reading frame lacked homology to any sequence in the nr (non-redundant protein) database (E-value > 10^5^).

To obtain independent rumen virome contigs the reads from [8] were also assembled using Gossamer [30] with K-mer = 60.

### Identifying integrated phage

Reads from [15] (microbiome sequence from the same samples as the 12 co-habited viromes) were aligned to each of the 14 largest contigs annotated in Figure 1 using BWA [32]. The proportion of alignments was calculated as A/B, where A is the number of reads that aligned to that contig, and B is the number of reads that aligned to all 14 contigs. The same procedure was applied to the virome data. Differences between the proportion of reads that aligned from the virome and microbiome were assessed by a paired t-test with two tails. A Bonferroni correction was applied by multiplying each p-value by the number of tests (14). To ensure that the overrepresentation in the microbiome was not due to a single conserved gene, alignments from the microbiome on the two significant contigs were manually checked. Converge of the contigs was relatively even, suggesting that the entire sequence was over represented in the microbiome (compared to expected contamination proportions).

### Richness

Overlapping reads were joined together using FLASH [33]. To estimate the number of species present in each of our samples, we used CatchAll [13], employing the minimo assembler within Circonspect [34] (the parameters changed from default were -u 20 -v 100 -r 1 -l 35 -s 100000) on 100000 joined reads.

### Between sample comparison

To compare the abundance of each contig in each sample the sequence reads were mapped to the assembled contigs using BWA-backtrack [32], with no insertions/deletions permitted. To compare the abundance of KEGG pathways we used 100,000 joined reads per sample and preformed a BLASTx search of the nr (non redundant protein) database of reads. Of the reads, 16.4% had a hit to the database with extremely relaxed parameters (e-value < 0.02). We then used MEGAN [35,36] (default parameters) to extract KEGG assignments form the BLAST output.

## Competing interests

The authors declare no competing interests.

## Author’s information

Steve Petrovski is currently at Peter MacCallum Cancer Centre, Melbourne Australia.

## Authors’ contributions

The parent study was run by PM. Samples were collected by PM and ER. Laboratory work was completed by SP. Analysis was performed and the manuscript written by ER. Interpretation of results, critique and final editing of the manuscript was performed by ER, SP, PM and BH. All authors have seen and approved the manuscript.
